# Effect of In Ovo Vitamin C Injection against Mobile Phone Radiation on Post-Hatch Performance of Broiler Chicks

**DOI:** 10.3390/vetsci9110613

**Published:** 2022-11-05

**Authors:** Fatma Yenilmez

**Affiliations:** Plant and Animal Production Department, Vocational School of Tufanbeyli, Cukurova University, Adana 01640, Turkey; fyenilmez@cu.edu.tr; Tel.: +90-322-338-7784

**Keywords:** in ovo, mobile phone radiation, vitamin C, performance, blood metabolites

## Abstract

**Simple Summary:**

The use of mobile phones has become globally widespread and a dependance on them has also increased in recent years. Therefore, the harmful effects of the electromagnetic waves emitted by mobile phones and base stations on humans, animals, and other living organisms have been the subject of many studies. The current study aimed to investigate the effect of in ovo injection of vitamin C to reduce the harmful effects of electromagnetic waves emitted from mobile phones on chicken embryos. In this context, a total of 750 fertilized eggs were exposed to electromagnetic waves for 1050 min during the incubation period. On the 17th day of incubation, vitamin C was injected into the amnion sac of the embryos, and hatched chicks’ performance was observed for 5 weeks after hatching. In ovo vitamin C injection improved the post-hatch performance of chicks but increased the oxidative damage in broiler chicks. The results showed that in ovo vitamin C injection can be a tool to improve carcass weight, production efficiency, and mortality, eliminating the possible negative effects of exposure to electromagnetic waves in the embryonal period on performance. However, the results of the study need to be supported by further investigation.

**Abstract:**

This study aimed to investigate the effect of in ovo injection of vitamin C to reduce the harmful effects of electromagnetic waves (EMWs) emitted from mobile phones on chicken embryos. In this study, a total of 750 fertilized eggs of Ross 308 were exposed to EMWs for 1050 min during the incubation period. On the 17th day of incubation, the eggs were divided into three groups and solutions were injected into the amnion sac of embryos. The chicks were housed separately in accordance with in ovo treatments for 5 wk after hatching. An in ovo vitamin C injection resulted in a lower hatching weight. The post-hatch mortality or production efficiency factor of birds in the in ovo vitamin C injection group and the intact egg group were comparable, and were better than that of the other negative control group. In ovo vitamin C injection in eggs subjected to EMWs significantly increased their body weight gain, carcass weight, abdominal fat weight, and AST levels, but reduced spleen weight and PON-1 levels. In conclusion, an in ovo vitamin C injection in eggs subject to mobile phone EMWs improved the post-hatch performance of chicks, but low PON and high AST activities indicate an increase in oxidative damage among broiler chicks.

## 1. Introduction

Presently, mobile phone use is widespread in Turkey (ranking eight in the world for mobile phone use) and globally, and they are used for many purposes. They are also widely used by those working in poultry houses, as in every field. With the widespread use of mobile phones, the amount and duration of radiation we are exposed to have increased.

The harmful effects of the electromagnetic waves (EMWs) emitted by mobile phones and base stations on humans, animals, and other living organisms have been the subject of many studies in recent years. Prolonged exposure to mobile phones that emit low-intensity radiation and generate nonionizing electromagnetic fields has effects on DNA and cells [1]. It may cause the onset of cancer in cells and tissues [2] and negatively affect sperm motility and morphology [3]. Most research in this field has focused on the damage caused by EMWs. Based on various reports on chickens, it has been found that EMWs affect embryo cell proliferation and liver [4] and embryo development [5]. Various investigations have revealed the harmful effects of exposure to radiation on embryos, including increased embryo mortality [6] and decreased amount of shell breakage and embryo hatching [7]. Similarly, there have been observations of abnormal embryonal developmental eye enlargement and brain defects [8]. The effect of radiation on biological tissues is direct (thermal) or through free radicals (nonthermal). Exposure to radiation increases the concentration of free radicals in tissues and causes oxidative stress [9]. Free radicals and oxidative stress play important roles in the emergence of many important diseases such as cancer, autoimmune disorders, rheumatoid arthritis, and cardiovascular and neurodegenerative diseases [10]. The antioxidant defense system keeps the formation of free radicals under control and prevents their harmful effects. However, when the concentrations of free radicals increase too much, the antioxidant defense system becomes insufficient and cannot prevent their harmful effects [9]. In this case, external antioxidant supplementation for animals is of great importance. Antioxidants react to free radicals, stabilize them, and prevent their harmful effects. Therefore, numerous studies have focused on the powerful antioxidant role of vitamin C, which plays a role in the development and strengthening of bones, teeth, gums, skin, joints, and wound healing [11]. It has been reported that vitamin C supplementation has a protective effect through a buffering role against free radicals emerging after radiation [12]. On the other hand, various studies have shown that vitamin C has a protective effect against gamma-ray intestinal damage, computer radiation, and electromagnetic radiation against kidney damage [13,14].

Research on in ovo injection has been increasing in recent years. In this method, a liquid solution comprising various additives, such as protein, vitamins, minerals, hormones, and antibodies are injected into the embryo, embryonic sacs, or the air space of the egg in any period of incubation. This practice makes the vaccination of the embryo during the incubation period possible, and can ensure intestinal development [15], stimulate the developing immune system, enhance post-emergence immunity [16,17], reduce illness and mortality [18], and improve of muscle development and body weight [19].

It was hypothesized that the EMWs emitted by mobile phones may damage embryo post-hatch development, performance, and carcass characteristics due to a reduction in chick immunity. As far as we know, there is no information currently available on the in ovo injection of vitamin C as a defense against mobile phone radiation in eggs. For this reason, in this study, we aimed to investigate the in ovo injection of vitamin C on post-hatch performance and response among EMW-challenged broiler eggs.

## 2. Materials and Methods

The present study was carried out in the Poultry Unit of the Agriculture Faculty of Cukurova University (Latitude: 37.033955 and Longitude: 35.347813). All the protocols used in this experiment were approved by the Cukurova University Animal Experiments Local Ethics Committee.

### 2.1. Incubation

A total of 750 fresh hatching eggs (Ross 308) were provided from a commercial flock at 40 weeks of age for use in this study. Eggs of similar weight were randomly divided into 3 treatment groups with 250 eggs per treatment, with 5 replicates of 50 eggs each. Standard incubation procedures were applied until hatching (dry-bulb temperature of 37.5 °C; 55–60% relative humidity in the developmental part until 19th day of incubation; eggs turned once per hour during the first 18 days; 37.2 °C temperature and 70–75% relative humidity from day 19 until hatching).

### 2.2. Radiation Application 

Samsung Galaxy Ace 2 18,160 mobile phones with a dual-core 800 MHz and a SAR (specific absorption rate) value of 0.52 W/kg for the head and 0.76 W/kg for the body were placed in the middle part of the incubators (3 trays on the top and 3 trays on the bottom). Care was taken to ensure that there was the same number of eggs from each group on each floor and that the distances of the eggs to the phone were equal. The distance of the phone to the nearest egg was 2 cm, and the farthest egg was 35 cm. The phone was muted, and a total of 1050 min of calls were made during a period of 21 days, comprising 10 calls a day with durations of 5 min each, totaling 50 min per 24 h. The time between calls has been released [4]. The eggs remained in an area where they were subjected to 2.46 ± 0.27–7.22 ± 1.03 V/m and a magnetic field of 0.0246–0.0722 µT. A GM3120 digital electromagnetic radiation measuring device was used to measure the radiation intensity.

### 2.3. In Ovo Injection 

A powder form of vitamin C was obtained from Shandong Luwei Pharmaceutical Co., LTD, originating in China. A measure of 33.3 g of vitamin C powder was added to 200 mL of deionized water and dissolved with the help of a mechanical mixer to prepare an injection solution.

The eggs were tested for fertility under lamp control and were cleaned and prepared for in ovo injection at room temperature. Fertile eggs were divided into 3 groups: A = negative control (no injection); B = positive control (deionized water = 0.6 mL); C = vitamin C (0.6 mL). On the 17th day of embryonic development [20], the thick part of the egg was sterilized with 70% ethanol. Using a 21-gauge needle, 31 mm was inserted into the amniotic fluid and 0.6 mL of the relevant solutions were injected [21].

### 2.4. Experiment Unit

The chicks (Ross 308) were housed separately in accordance with in ovo treatments for 5 weeks after hatching. The hatched chicks were weighed, and they were reared separately according to in ovo applications (3 main groups and 4 subgroups comprising 456 chicks) with a mixed male and female population. The experimental birds were housed in a floor pen measuring 4 (2 m × 2 m) square meters for 5 weeks and their performance parameters were recorded weekly. During the chick period, suitable feeders and drinkers were used for the chicks, then a hanging feeder and an automatic hanging drinker were placed in each compartment. All groups were fed with standard basal diet (Table 1). The starter feed (1–3 weeks; 230 g/kg HP and 3000 kcal/kg ME) and finisher feed (4–5 weeks; 200 g/kg HP and 3200 kcal/kg ME) was obtained from a commercial feed company. A continuous lighting program (24 h), 60 ± 70% controlled relative humidity, and ad libitum feed and water were supplied during the experiment.

### 2.5. Performance and Carcass Characteristics 

During the experiment period, body weights (BW) and feed intake (FI) were determined weekly. Feed–conversion ratio (FCR) was obtained by dividing total feed consumed by bodyweight gain. The mortality (dead chicks/housed chicks) was calculated as a percentage for the experiment overall. The production efficiency factor was calculated with the following formula [22].

$$\mathrm{PEF}=\;\frac{\mathrm{LW}\;\left(\mathrm{kg}\right)\;\times \;\mathrm{LA}\;\left(\%\right)}{\mathrm{SA}\;\left(\mathrm{days}\right)\;\times \;\mathrm{FCR}\;}\times 100$$

LW (kg) = Live weight,

LA (%) = Livability,

SA (days) = Slaughter age,

FCR = Cumulative feed intake (kg)/total weight gain (kg).

At the end of the experiment, a total of 96 birds, 32 birds from each experimental group (4 males and 4 females randomly selected from each subgroup) showing the average body weights of the group were slaughtered; the weights of the hot and cold carcass, heart, spleen, liver, pectoral muscle, testicles, and abdominal fat were determined. 

### 2.6. Blood Analyses 

Blood samples were collected from the neck during slaughtering for a total of 60 (20 × 3) animals, comprising 10 males and 10 females of each group. They were centrifuged for 10 min (2000× *g*) and plasma samples were preserved at −20 °C until analyses. Paraoxonase-1 (PON-1; U/L), total thiol (TTL; µmol/L), native thiol (NTL; µmol/L), disulfide, myeloperoxidase (MPO; U/L), aspartate aminotransferase (AST; U/L), and alanine aminotransferase (ALT; U/L) levels in the serum samples were analyzed using commercial kits (Baran Medical Inc., Ankara, Turkey).

### 2.7. Statistical Analyses 

The effect of gender on mortality rate, the production efficiency factor, the feed consumption, and the feed–conversion ratio were excluded from the statistical model due to the insignificance of gender [23]. The effects were considered significant at *p* < 0.05. The Duncan test was applied to analyze the pairwise differences among the least mean square values. Results were presented as the least mean square and standard error.

Yijk = µ + Fi + Tj + eijk

where Yijkl—response variable (e.g., body weight, carcass and internal organ weights, and blood metabolites of broiler chicks); µ—overall mean; Fi—fixed factorial effect of in ovo injection (A—negative control; B—positive control; C—vitamin C); Tj—fixed factorial effect of sex (M and F); Eijk—residual, assumed to be normally distributed.

## 3. Results

### 3.1. Body Weight

The results obtained in the experiment showed that in ovo injection or sex of birds had significant effects on BW (Table 2). The group with vitamin C injection had the lowest BW at hatching; however, the same group soon showed higher BW than the other groups in the following weeks. Male birds gained higher BW than females at the end of the experiment (2077 vs. 1767).

### 3.2. Post-Hatch Mortality and Production Efficiency Factor

It was shown that the mortality and production efficiency factor in negative control or vitamin C treatments were greater than that of the distilled water treatment (*p* < 0.01) (Table 3).

### 3.3. Feed Intake

Weekly feed intake results are presented in Table 4. The negative control and vitamin C groups were significantly affected by the feed intake at the 1st (0.012), 4th, and 5th weeks (0.044, 0.030) of the experiment. Although there was no difference between the individual feed intakes of the treatment groups, group B was found to be the lowest for feed intake (2329.6 g) and group C was observed to be the highest feed intake (2763.5 g) at the end of the experiment.

### 3.4. Feed–Conversion Ratio

FCR showed an improving trend towards improvement with the injection of vitamin C (*p* = 0.06) at the end of the trial, although the difference between the groups was not significant (Table 5).

### 3.5. Carcass and Internal Organs

Hot or cold carcass weight was affected by in ovo vitamin C injection and the sex of the birds (Table 6). 

Pectoral muscle, heart, and liver weights were significantly related to the sex of the birds. The spleen weight of the vitamin C group was lower than in the control groups, and was affected by the sex of the birds. Abdominal fat weight was significantly increased among the injected groups when compared with the intact group (*p* = 0.05); however, it was not influenced by the sex of the birds (*p* > 0.05). The testicular weight of all groups was found to have similar mean weight at the end of the experiment (*p* > 0.05). 

### 3.6. Blood Metabolites

No significant differences were observed for serum TTL, NTL, MPO, ALT, and disulfide concentration (Table 7). However, serum PON-1 and AST concentration were influenced (Table 6; *p* < 0.01, *p* = 0.05, respectively) by the in ovo injection of the birds. 

## 4. Discussion

Numerous researchers have investigated the effects of mobile phone EMWs exposure on body weight gain and growth performance [7,8,24]. For example, Siddiqi et al. [4] found significant growth retardation on the 10th day in an experimental group exposed to EMWs compared with the control group. They found no significant growth differences on the 5th or 15th day, and they did not encounter any deaths. Similarly, Pawlak et al. [7] in their study on the effect of EMWs with a frequency of 900 MHz on the hatchability of chicken embryos; reported that the exposure did not affect the body weight of chicks. 

Previous research has shown that vitamin C has a protective effect against radiation [12,25] and there is evidence that the in ovo injection of vitamin C gives positive results. In a study, Selim et al. [26] conducted in ovo vitamin C and E injections on fertilized duck eggs, which were found to increase the body weights of duck chicks at the end of the experiment. In another study, vitamin A, B1, B6, C, and E were injected into broiler breeder eggs; vitamin A (100 UI) and C (50 mg) were found to increase the hatching weight [27]. In the same direction, Zhang et al. [19] investigated the in ovo administration of L-ascorbic acid (AA) on broiler performance, carcass characteristics, antioxidant status of plasma, and quality of meat. It was determined that AA injection (3–12 mg) had lasting positive effects on chick growth and leg muscle development. They found that injecting higher AA dosages (36 mg) could have the potential to improve broiler meat quality. However, generally (from day 0 to day 45), increasing in ovo injected AA dosages did not cause any significant treatment differences for body weight at day 45. A similar study evaluated the possibility of in ovo injection of vitamin C and E to protect chicks from hatching-sourced oxidative stress [28]. As a result, vitamin C and E injection was found to be an effective method for protecting newly hatched chicks from lipid peroxidation and the negative effects of heat stress in early life, and was found to strengthen the antioxidant defense system. In another study, Zhang et al. [29] emphasized that AA has the potential to stimulate growth. When our research results were examined, it was seen that the weight of the vitamin C group was high during the 5 weeks. El-Kholy et al. [30] reported that in ovo injection of vitamin C into Japanese quails eggs increased the body weight gain of the birds. However, Soltani et al. [31] did not observe differences in the hatchability, post-hatch performance, or blood parameters after in ovo injection of different AA levels. Similarly, Ghane et al. [32] and Zhu et al. [33] stated that in ovo vitamin C has no significant effect on body weight. Contrary to the existing literature, we determined that the application of vitamin C positively affected the weekly body weight and slaughter weight (5th week), and increased the FI and consequently the weight gain of broilers. Altan et al. [28] showed that mortality in chicks was not affected by in ovo injection of vitamin C and E. In the current study, in ovo vitamin C administration positively affected the mortality of chicks. In this study, in ovo injection of vitamin C against EMWs significantly increased the production efficiency factor, so could be used as an alternative for profitable production. On the other hand, in this study, no significant impact of in ovo injection of vitamin C on FCR could be found, which seemed parallel to the previous research results [31,33]. In contrast with the results of this study, Zhang et al. [19] determined that in ovo administration of L-ascorbic acid (3–12 mg) had lasting positive effects on the growth of chickens. In general (from day 0 to day 45), increasing in ovo-injected AA dosages significantly increased the FCR (*p* = 0.045). The effects of in ovo feeding of vitamin C in a different study on FCR may depend on the dose and source. 

According to the research results, body weight was affected by the sex of the birds and male birds gained higher body weight than females at the end of the experiment. Parallel to this, hot or cold carcass weight was also affected by sex and was higher among males. Pectoral muscle, heart, and liver weights were found to be significantly related to the sex of the birds and higher in males, similarly to the hot and cold carcass weights. Similarly, spleen weight was affected by the sex of birds and was higher in males, but the abdominal fat weight was not influenced. Selim et al. [26] indicated that in ovo injection of either vitamin E or ascorbic acid had no significant effect on carcass characteristics in Muscovy ducks.

Research has proved that in ovo administration of vitamin C or vitamin E into amnion fluid does not affect carcass characteristics of broiler chicks [34]. Similarly, Zhang et al. [19] did not determine differences in percentages of breast, wing, or abdominal fat of carcasses after ovo injection of L-ascorbic acid. Contrary to these studies, in the current study, in ovo vitamin C injection as a defense against EMWs was found to increase the hot and cold carcass, pectoral muscle, heart, liver, and abdominal fat weights. Zhu et al. [35] stated in their study that spleen development and maturation were regulated by in ovo feeding of vitamin C. However, in this study, the fact that in ovo vitamin C supplementation caused a decrease in spleen weight can be explained by the atrophy of the spleen due to oxidative stress, which is related to rapid growth or exposure to EMWs during the incubation period. The spleen in adult birds is the largest immune organ and is characterized as an organ that produces predominantly lymphocytes and destroys erythrocytes [33,36]. It has been stated that a decrease in PON-1 activity occurs in cases of high oxidative stress states, such as metabolic syndrome, obesity, uncontrolled diabetes, cardiovascular disorders, and fatty liver disease [37,38,39]. The low PON-1 level in group C may be due to cardiovascular disorders that are associated with rapid growth or fatty liver. It was speculated that the decreased PON-1 level may increase the intensity of stimuli sent from oxidative free radicals during the filtration of the spleen. However, spleen weight in chicks was decreased by in ovo administration of vitamin C in defense against EMWs. 

Vitamin C reacts to the free radicals that arise as a result of radiation, stabilizes them, and acts as a buffer against post-oxidation cell damage [12,25] and thus promotes growth. For this reason, in ovo administration of vitamin C had lasting positive effects on the body weights of the chickens in this study. The decrease in PON-1 concentration can be interpreted as a weakening of the defense system against the rapid body weight gain of the birds. The other most common indicator used to describe the physiological state of the body is hematological indices [24]. AST and ALT synthesized in the intracellular environment reach high concentrations in serum by passing into the blood as a result of the change in the permeability of the cell membrane or cell disintegration. It has been reported that the increase in these enzyme activities is accepted as the most important indicator of liver damage [40].

In the present study, based on the literature, embryos were exposed to the negative effects of radiation. However, a control group that was not exposed to mobile phone radiation was not used. If such a group was formed, the negative effects of radiation could be observed comparatively, and the effects of vitamin C could be revealed more clearly. This may be taken into account in further studies.

## 5. Conclusions

In defense against the harmful effects of EMWs applied to fertilized broiler eggs during the incubation period, in ovo vitamin C injection was found to positively affect the growth performance, mortality, and production efficiency factor of the chicks. In addition, the hot and cold carcass weights increased and spleen weight decreased. The high AST level and low PON-1 activity in the C group support the idea that liver damage caused by oxidative stress was associated with increased body weight and carcass weight, as seen in the vitamin C injection group compared with the other groups.

As a result of this study, in ovo vitamin C injection can be a tool to improve the carcass weight, production efficiency, and mortality of chickens, eliminating the possible negative effects of exposure to EMWs during the embryonal period on performance. However, the results of the study need to be supported by further investigation.

## Figures and Tables

**Table 1:** Standard basal diet.

Ingredients %	Starter (1–3 Weeks)	Finisher (4–5 Weeks)
Maize	52.16	58.58
Soybean meal (46% CP)	9.84	5.62
Full fat soybean	27.23	22.25
Wheat middlings	3.00	5.00
Corn gluten meal (60% CP)	2.29	2.00
Sunflower meal (34% CP)	2.00	4.00
Dicalcium phosphate	1.03	0.53
Limestone	0.94	0.75
Lysine sulfate	0.44	0.40
DL-methionine	0.29	0.21
Salt	0.25	0.23
Sodium sulfate	0.13	0.10
Thereonine	0.11	0.05
Mineral Premix ^1^	0.10	0.10
Vitamin Premix ^2^	0.10	0.10
Choline-60	0.05	0.05
Anticoccidial	0.05	0.05
Calculated Analysis, %		
Dry matter	87.97	88.43
Crude Protein	23	18–19
Crude Fiber	3–4	3–4
Crude Fat	4–5	6–7
Crude Ash	5–6	4–5
Starch	33–34	38–39
Calcium	0.88	0.67
Total P	0.73	0.62
Sodium	0.18	0.16

^1^—each 2 kg aliquot of vitamin premix contains 12,000,000 IU vitamin A, 3,500,000 IU vitamin D3, 100 g vitamin E, 3 g vitamin K_3_, 2.5 g vitamin B1, 6 g vitamin B_2_, 25 g niacin, 12 g Ca-D-pantothenate, 4 g vitamin B_6_, 15 mg vitamin B_12_, 1.5 g folic acid, 150 mg D-biotin, 100 g vitamin C, and 450 g choline chloride; ^2^—each kg of mineral premix contains 100 mg manganese, 25 g iron, 65 g zinc, 15 g copper, 0.25 g cobalt, 1 g iodine, and 0.2 g selenium.

**Table 2:** The effects of in ovo injection of vitamin C and sex on body weight (g).

Weeks	In Ovo Injection				Sex			*p*	
	A	B	C	SEM	M	F	SEM	In Ovo	Sex
IBW	40.2 ^b^	40.9 ^a^	39.9 ^c^	0.15	40.6	40.1	0.119	<0.01	0.015
1	139.5 ^b^	125.9 ^c^	155.9 ^a^	3.69	142.4	138.4	2.44	<0.01	0.295
2	388.7 ^b^	359.7 ^b^	421.5 ^a^	9.76	410.5	369.4	7.37	<0.01	<0.01
3	774.6	730.4	875.5	35.49	826.7	760.3	26.77	0.058	0.122
4	1324.2	1253.2	1390.5	48.16	1419.4	1225.9	36.34	0.291	<0.01
5	1890.2	1828.1	2048.9	75.27	2077.7	1767.1	57.19	0.056	<0.01

^a,b,c^—the difference between the group means indicated with different letters in the same column is statistically significant (*p* < 0.05); **SEM**—standard error of the mean; **IBW**—initial body weight; **A**—negative control; **B**—positive control; **C**—vitamin C; **M**—male; **F**—female.

**Table 3:** The effects of in ovo injection of vitamin C on post-hatch mortality and production efficiency factor (%).

Parameters	In Ovo Injection				*p*
	A	B	C	SEM	
**Post-hatch mortality rate**	5.3 ^b^	14.3 ^a^	3.3 ^b^	1.98	0.019
**Production Efficiency Factor**	312.9 ^a^	220.1 ^b^	369.0 ^a^	21.56	<0.01

^a,b^—the difference between the group means indicated with different letters in the same column is statistically significant (*p* < 0.05); **SEM**—standard error of the mean; **A**—negative control; **B**—positive control; **C**—vitamin C.

**Table 4:** The effects of in ovo injection of vitamin C on feed intake (g).

Weeks		In Ovo Injection				*p*
		A	B	C	SEM	
Group level feed intake	1	3419.2 ^a^	2580.8 ^b^	3361.7 ^a^	146.46	0.012
	2	14,036.5	11,720.6	14,085.2	687.93	0.084
	3	34,884.2	25,133.6	32,947.5	2391.73	0.060
	4	63,166.7 ^a^	43,241.6 ^b^	60,346.2 ^a^	4589.04	0.044
	5	95,066.5 ^a^	64,492.6 ^b^	93,445.7 ^a^	6666.44	0.030
IFI		2624.5	2329.6	2763.5	107.91	0.072

^a,b^—the difference between the group means indicated with different letters in the same column is statistically significant (*p* < 0.05); **SEM**—standard error of the mean; **A**—negative control; **B**—positive control; **C**—vitamin C; **IFI**—mean of the individual feed intake.

**Table 5:** The effects of in ovo injection of vitamin C on the feed–conversion ratio.

Weeks	In Ovo Injection				*p*
	A	B	C	SEM	
1	0.9	0.8	0.9	0.03	0.266
2	1.1 ^b^	1.2 ^a^	1.1 ^b^	0.03	0.055
3	1.3	1.3	1.2	0.03	0.142
4	1.4	1.4	1.3	0.03	0.145
5	1.5	1.5	1.4	0.02	0.068

^a,b^—the difference between the group means indicated with different letters in the same column is statistically significant (*p* < 0.05); **SEM**—standard error of the mean; **A**—negative control; **B**—positive control; **C**—vitamin C.

**Table 6:** The effects of in ovo injection of vitamin C and sex on carcass and internal organ weights of broiler chicks at market age (day 42).

Parameters	In Ovo Injection				Sex			*p*	
	A	B	C	SEM	M	F	SEM	In Ovo	Sex
Hot carcass weight (g/bird)	1443.9 ^b^	1392.8 ^b^	1548.7 ^a^	25.43	1546.1	1377.5	20.73	0.046	<0.01
Cold carcass weight (g/bird)	1428.4 ^b^	1378.6 ^b^	1531.9 ^a^	24.69	1528.9	1363.6	20.13	<0.01	<0.01
Pectoral muscle weight (g/bird)	227.2	222.5	239.6	6.16	243.8	215.7	5.03	0.147	<0.01
Heart weight (g/bird)	12.0	11.5	12.3	0.35	13.0	10.9	0.28	0.267	<0.01
Liver weight (g/bird)	43.7	43.7	45.6	1.24	47.0	41.7	1.00	0.522	<0.01
Spleen weight (g/bird)	3.1 ^ab^	3.3 ^a^	2.8 ^b^	0.15	3.3	2.9	0.12	0.049	0.020
Abdominal fat weight (g/bird)	19.9 ^b^	22.9 ^ab^	24.4 ^a^	1.36	23.1	21.7	1.11	0.052	0.354
Testicular weight (g/bird)	0.4	0.4	0.4	0.03	-	-	-	0.599	-

^a,b^—the difference between the group means indicated with different letters in the same column is statistically significant (*p* < 0.05); **SEM**—standard error of the mean; **A**—negative control; **B**—positive control; **C**—vitamin C; **M**—male; **F**—female.

**Table 7:** The effects of in ovo injection of vitamin C and sex on blood metabolites of broiler chicks at market age (day 42).

Parameters	In Ovo Injection				Sex			*p*	
	A	B	C	SEM	M	F	SEM	In Ovo	Sex
PON-1(U/L)	12.2 ^ab^	16.6 ^a^	10.2 ^b^	1.49	14.3	11.8	1.22	0.012	0.156
TTL(µmol/L)	819.9	794.6	720.2	33.46	755.0	801.5	27.40	0.100	0.237
NTL(µmol/L)	199.7	241.3	211.6	23.62	204.0	231.0	19.34	0.446	0.329
Disulfide	310.1	276.7	254.3	18.87	275.5	285.2	15.46	0.119	0.660
MPO (U/L)	70.5	48.3	49.3	11.90	58.0	54.0	8.74	0.336	0.773
AST (U/L)	353.1 ^b^	358.7 ^b^	409.2 ^a^	17.23	369.3	378.0	14.10	0.047	0.666
ALT (U/L)	10.6	9.6	9.3	0.95	9.1	10.6	0.78	0.619	0.181

^a,b^—the difference between the group means indicated with different letters in the same column is statistically significant (*p* < 0.05); **SEM**—standard error of the mean; **A**—negative control; **B**—positive control; **C**—vitamin C; **M**—male; **F**—female.

## Data Availability

Data will be available upon request.
